# The Clinical Features of Gas Poisoning

**Published:** 1903-06-27

**Authors:** 


### THE CLINICAL FEATURES OF GAS POISONING.

In America, where "water-gas" is much used, cases of poisoning have increased very largely of late years, and in 1901 as many as 156 deaths were referred to this cause in New York. A considerable number came under the observation of Dr. P. M. Pilcher while acting as resident of one of the Brooklyn hospitals, and he made a clinical study of them the subject of a recent communication to the Medical Society of the County of Kings. In 25 cases, of which details were given, five were cases in which exposure to gas had been short and recovery was uneventful, but six resulted in death. The more serious cases were those in which the gas had been inhaled during sleep, and in five of the fatal cases the exposure had lasted at least 8 hours before the patients were discovered and removed. One died 18 hours after admission, four survived for about 30 hours, and one did not die until the sixth day. Of the six deaths, in five cases the immediate cause of the fatal issue was pulmonary œdema. Only one of the patients who recovered was unconscious for more than a few hours, while in half of those who died coma persisted until the end, the others becoming sufficiently conscious to speak. In all the cases besides cyanosis and the ordinary appearances of more or less complete asphyxia, there was with one exception a considerable rise of temperature, and rapidity of pulse and respiration. In the exception, the temperature was subnormal, but pulse and respiration corresponded with the other cases. In the fatal cases the rise of temperature kept step with the development of the pulmonary œdema, and in four of them rose to over 107° Fahr. Clinically, the cases fell roughly into three groups: mild cases of temporary asphyxia, profound cases of general poisoning, and those in which œdema of the lungs had already developed when the patients were found and brought under treatment. To these a fourth class might be added in which death has already occurred or is occurring from pure carbo-monoxide poisoning. In all these classes of recoverable cases the indications for treatment are numerous. In the mild cases in which there is simply coma without marked alteration in temperature and practically normal respiration and heart action, fresh air, heart stimulants, and protection from cold are all that is necessary to ensure return to consciousness in from a few minutes to one or two hours. In the more severe cases the conditions to be met are (1) the blood changes due to the formation of carbo-monoxide hæmoglobin in greater or smaller quantity; (2) the exhaustion of vital nerve centres due partly to the lack of oxygen and partly to the presence of carbo-monoxide; leading to coma, decrease of blood pressure and disturbance of the respiratory cardiac, and heat regulating functions of the central nervous system. There is also a loss of muscular tone which, from the delay in post-mortem changes in fatal cases, appears to be due to the direct action of C.O. in the tissues. As a consequence of the exhaustion of the central nervous system, the vascular paresis is great, resulting in visceral and cerebral congestion. The tendency to develop œdema of the lungs is marked, and probably due to direct irritation, and cardiac failure, most marked in the left ventricle, results in congestion in, and subsequent exudation from, the lung capillaries. It often develops with very great rapidity, and in cases which end fatally after recovery from coma it is quite the commonest cause of death. In treatment, cardiac stimulants are called for, but oxygen, unless the patient can be forced to inhale it pure, is not of marked utility. The difficulty is to break up the C.O. hæmoglobin. Free venesection followed by transfusion of normal salt solution seemed to do more good in this direction than anything else, and direct transfusion of blood is certainly indicated. The lung œdema is best met by free cupping and mustard stupes to the chest and strong purges such as croton oil.

